# Multi-omics profiling reveals dysregulated ribosome biogenesis and impaired cell proliferation following knockout of *CDR2L*

**DOI:** 10.1186/s12885-024-12399-z

**Published:** 2024-05-27

**Authors:** Eirik Tveit Solheim, Yola Gerking, Torbjørn Kråkenes, Ida Herdlevær, Even Birkeland, Cecilie Totland, Fiona Dick, Christian Alexander Vedeler

**Affiliations:** 1 Department of Clinical Medicine, University of Bergen, Bergen, Norway; 2 Neuro-SysMed - Centre of Excellence for Experimental Therapy in Neurology, Departments of Neurology and Clinical Medicine, Bergen, Norway; 3 Department of Neurology, Haukeland University Hospital, Bergen, Norway; 4 Norwegian Institute of Public Health, Bergen, Norway

**Keywords:** Paraneoplastic cerebellar degeneration, PCD, CDR2L, Ovarian cancer, Ribosome dysfunction, Multi-omics, Knockout

## Abstract

**Background:**

Cerebellar degeneration-related (CDR) proteins are associated with paraneoplastic cerebellar degeneration (PCD) – a rare, neurodegenerative disease caused by tumour-induced autoimmunity against neural antigens resulting in degeneration of Purkinje neurons in the cerebellum. The pathogenesis of PCD is unknown, in large part due to our limited understanding of the functions of CDR proteins. To this end, we performed an extensive, multi-omics analysis of *CDR*-knockout cells focusing on the CDR2L protein, to gain a deeper understanding of the properties of the CDR proteins in ovarian cancer.

**Methods:**

Ovarian cancer cell lines lacking either CDR1, CDR2, or CDR2L were analysed using RNA sequencing and mass spectrometry-based proteomics to assess changes to the transcriptome, proteome and secretome in the absence of these proteins.

**Results:**

For each knockout cell line, we identified sets of differentially expressed genes and proteins. *CDR2L*-knockout cells displayed a distinct expression profile compared to *CDR1*- and *CDR2*-knockout cells. Knockout of *CDR2L* caused dysregulation of genes involved in ribosome biogenesis, protein translation, and cell cycle progression, ultimately causing impaired cell proliferation in vitro. Several of these genes showed a concurrent upregulation at the transcript level and downregulation at the protein level.

**Conclusions:**

Our study provides the first integrative multi-omics analysis of the impact of knockout of the *CDR* genes, providing both new insights into the biological properties of the CDR proteins in ovarian cancer, and a valuable resource for future investigations into the CDR proteins.

**Supplementary Information:**

The online version contains supplementary material available at 10.1186/s12885-024-12399-z.

## Introduction

Paraneoplastic neurological syndromes are rare, immune-mediated disorders triggered by cancer [1]. These syndromes are characterized by circulating antibodies directed against antigens expressed by neurons and cancer cells. Paraneoplastic cerebellar degeneration (PCD) is one of the most common forms of paraneoplastic neurological syndromes [2, 3]. It presents with subacute onset of limb and truncal ataxia, dysarthria, and nystagmus [4]. In patients with PCD, the dominant autoantibody detected in serum and cerebrospinal fluid is anti-Yo which is most frequently seen in patients with breast, ovarian or other gynaecological cancers [2, 4]. Anti-Yo targets three intracellular antigens expressed by Purkinje neurons and cancer cells – the cerebellar degeneration-related (CDR) proteins CDR1, CDR2, and CDR2-like (CDR2L) [5–8]. The interactions between anti-Yo and CDR proteins have been shown to cause Purkinje neuron death [9, 10].

We demonstrated recently that CDR2L is likely the major antigen of anti-Yo [11]. Increased expression of *CDR2L* mRNA has been observed in ovarian tumours from PCD patients with anti-Yo antibodies compared to ovarian tumours from patients without PCD or anti-Yo antibodies [12]. Due to our limited understanding of the biological functions of the CDR2L protein, the consequences of this increased expression in tumours are not known. Previous studies showed that CDR2L localizes to the cell cytoplasm in association with membrane-bound and free ribosomes in both Purkinje neurons and cancer cells [13–15]. Further, we demonstrated that CDR2L interacts with several ribosomal proteins, suggesting a role in protein synthesis [15].

In the present study, we used a multi-omics approach to explore changes in the transcriptome, proteome, and secretome induced by knockout of *CDR1*, *CDR2*, and *CDR2L* in ovarian cancer cells. Since CDR2L is considered the major antigen in Yo-associated PCD, we characterized the effects of *CDR2L* knockout in detail. We found that loss of CDR2L had a distinct effect on the transcriptome and proteome compared to loss of CDR1 or of CDR2. Following knockout of *CDR2L*, genes associated with ribosome biogenesis, protein translation and cell cycle progression were downregulated, and cell proliferation was impaired. This study provides an extensive analysis of *CDR*-knockout cells and offers a valuable resource for future investigations into the CDR proteins.

## Results

### Knockout of genes encoding CDR proteins induces expression changes in the transcriptome, proteome, and secretome of ovarian cancer cells

**Fig. 1:**
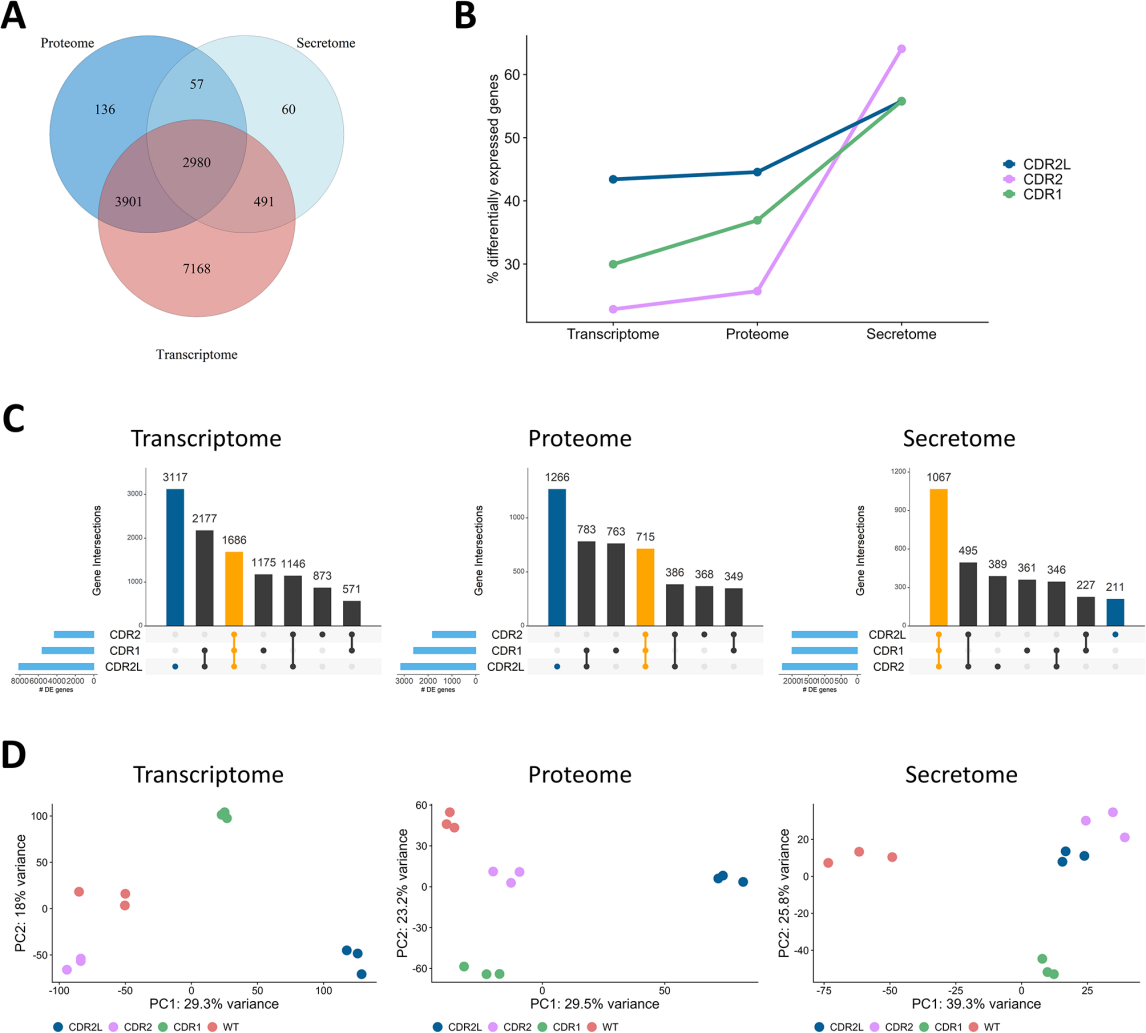
Comparison of changes induced by knockout of *CDR1*, *CDR2*, and *CDR2L* in the transcriptome, proteome, and secretome of ovarian cancer cells. **(A)** Venn diagram showing the number of genes identified in each dataset and the overlap between datasets. **(B)** The proportion of differentially expressed genes (y-axis) for each knockout cell line (colour) per dataset (x-axis). The percentage was calculated based on the number of genes in each pre-filtered dataset. **(C)** Upset plots where the rows correspond to the significantly (FDR < 0.05) differentially expressed genes in each knockout cell line, and the columns correspond to the intersecting genes in each dataset (panels). The orange bar highlights the number of differentially expressed genes common to all cell lines. The dark blue bar highlights differentially expressed genes unique to *CDR2L*-knockout cells. **(D)** Principal component plot. Datapoints represent samples in the first (x-axis) and second (y-axis) principal component space. Color-coding indicates group variable. Analysis was performed separately on each dataset (panels)

Using differential expression analysis, we investigated changes induced by knockout of *CDR1*, *CDR2*, and *CDR2L* in the transcriptome, and the proteomes of cell lysate and conditioned cell medium. These three omics datasets were separately normalized and subjected to pre-filtering, resulting in 18,719 genes in the transcriptomic dataset (Table S1): the majority of which were protein-coding (80.2%). There were 7,074 proteins in the cell lysate proteomics dataset (referred to as the proteome; Table S2) and 3,588 in the cell medium proteomics dataset (referred to as the secretome; Table S3). Comparison of the transcriptomics and proteomics data revealed that 6,881 (97.3%) and 3,470 (96.7%) proteins in the proteome and secretome datasets, respectively, were matched with protein-coding mRNAs (Fig. 1A). There were significant positive correlations between the transcriptome and proteome (Spearman’s rank correlation coefficient across wild-type and knockout cell lines, ρ = 0.53, *p*-value < 2.2e-16), between transcriptome and secretome (ρ = 0.38, *p*-value < 2.2e-16), and between proteome and secretome (ρ = 0.45, *p*-value < 2.2e-16; Figure S1).

Proteins present in the secretome include secreted proteins, proteins shed by membrane vesicles, and proteins leaked from dying cells. To assess the sources of proteins identified in the secretome, we compared these proteins with proteins in the Plasma Proteome Database [16], which are reportedly present in the plasma and serum, and with those in the Vesiclepedia Database [17], which have been detected in extracellular vesicles. Of the 3,588 proteins in the secretome, 55.8% were found in the Plasma Proteome Database and 86.3% were found in the Vesiclepedia database, suggesting that most of proteins in the secretome were derived from extracellular vesicles.

We performed differential expression analysis on each dataset comparing knockout cells to the wild-type (WT) cells, resulting in differentially expressed (DE) genes and proteins in the transcriptome, proteome, and secretome for each knockout cell line (Table 1). Effect sizes and significance values are listed in Tables S4-S6. There were more DE genes in the transcriptome in all group comparisons than in the proteome. Since mass spectrometry-based proteomics is less sensitive than RNA sequencing, resulting in fewer detected proteins than genes, the lower number of DE genes in the proteome compared to the transcriptome was not unexpected. The proportion of genes that were DE was similar between the transcriptome (mean proportion across all three knockout cell lines = 32.0%) and proteome (35.7%), whereas the secretome had a much higher proportion of DE genes (58.5%) with more than half of secreted proteins affected by the knockout, irrespective of CDR protein (Fig. 1B).

Some genes were DE in all three knockout cell lines. These constituted 15.7% of DE genes in the transcriptome and 15.4% of DE proteins in the proteome (FDR < 0.05 in all three knockout analyses, where the intersection was performed irrespective of direction of change). In the secretome, this proportion was more than twice as high with 34.4% of DE genes common to the three knockout cell lines (Fig. 1C). Over-representation analysis on these common DE genes was performed to identify the biological processes affected by the loss of any of the *CDR* genes (Table S7). In the transcriptome, cell adhesion, cell motility, extracellular structure organization, and negative regulation of nervous system development were among the enriched pathways. In the proteome, several metabolic processes were enriched, including organic hydroxy compound metabolic process, steroid metabolic process, and fatty acid metabolic process. In the secretome, ribosomes, RNA catabolic process, and protein localization to endoplasmic reticulum were enriched.

**Table 1:** Number of significantly differentially expressed genes (FDR < 0.05) in the transcriptome, proteome, and secretome of *CDR*-knockout cell lines versus wild-type cells

	CDR2L	CDR2	CDR1
**Transcriptome**			
Downregulated	4035 (21.6%)	2272 (12.1%)	2672 (14.3%)
Not significant	10,593 (56.6%)	14,443 (77.2%)	13,110 (70.0%)
Upregulated	4091 (21.9%)	2004 (10.7%)	2937 (15.7%)
**Proteome**			
Downregulated	1890 (26.7%)	1143 (16.2%)	1546 (21.9%)
Not significant	3922 (55.4%)	5256 (74.3%)	4462 (63.1%)
Upregulated	1262 (17.8%)	675 (9.5%)	1066 (15.1%)
**Secretome**			
Downregulated	1651 (46.0%)	1949 (54.3%)	1365 (38.0%)
Not significant	1587 (44.2%)	1289 (35.9%)	1586 (44.2%)
Upregulated	350 (9.8%)	350 (9.8%)	637 (17.8%)

### Knockout of *CDR2L* results in a unique transcriptomic and proteomic expression profile

To understand the differential effect of the knockout, we compared the number of DE genes between the three knockout cell lines. In the transcriptome, 43.4% of genes were DE in *CDR2L*-knockout cells compared to 22.8% in *CDR2*- and 30.0% in *CDR1*-knockout cells (Fig. 1C). In line with the higher proportion of DE genes, CDR2L also showed the highest number of uniquely altered genes (i.e., not significantly DE in the other knockout cell lines) with 29.0% of genes in the transcriptome uniquely altered in the *CDR2L*-knockout cells (Fig. 1C). Similarly, in the proteome, 44.6% of genes were DE in C*DR2L*-knockout cells, with 27.3% of genes in the proteome uniquely altered in C*DR2L*-knockout cells. The high number of uniquely altered genes in *CDR2L*-knockout cells suggests that loss of CDR2L has a distinct effect on the cells compared to loss of the other CDR proteins. In the secretome, however, most DE genes were common to all knockout cell lines, with *CDR2L*-knockout cells having the lowest number of uniquely altered genes.

Using principal component analysis on the three omic datasets separately, we observed a clustering of samples into the respective knockout groups, confirming a good accordance between biological replicates. The *CDR2L*-knockout cells were separated from the other cells, including WT, along the first principal component in the transcriptome and proteome but not in the secretome (Fig. 1D), further strengthening our hypothesis that loss of CDR2L has a unique effect inside the cell. We investigated this further using linear regression and found a significant association between the first principal component (representing the main axis of variation in the dataset) and the condition variable CDR2L versus the other cell lines for the transcriptome (*p*-value = 3.4e-04) and the proteome (*p*-value = 4.3e-08) but not for the secretome (*p*-value = 0.357). When comparing the 1000 genes with the highest absolute loadings in the first principal component, only 5.5% were common between the transcriptome and proteome, suggesting that the genes driving the separation between the *CDR2L*-knockout cell line and the other cell lines are different between the transcriptome and proteome. Based on these findings, we decided to further characterize the likely unique intracellular expression profile of the *CDR2L*-knockout cells.

### Gene set enrichment analysis reveals discordant changes in enriched pathways at transcript and protein level upon knockout of *CDR2L*

**Fig. 2:**
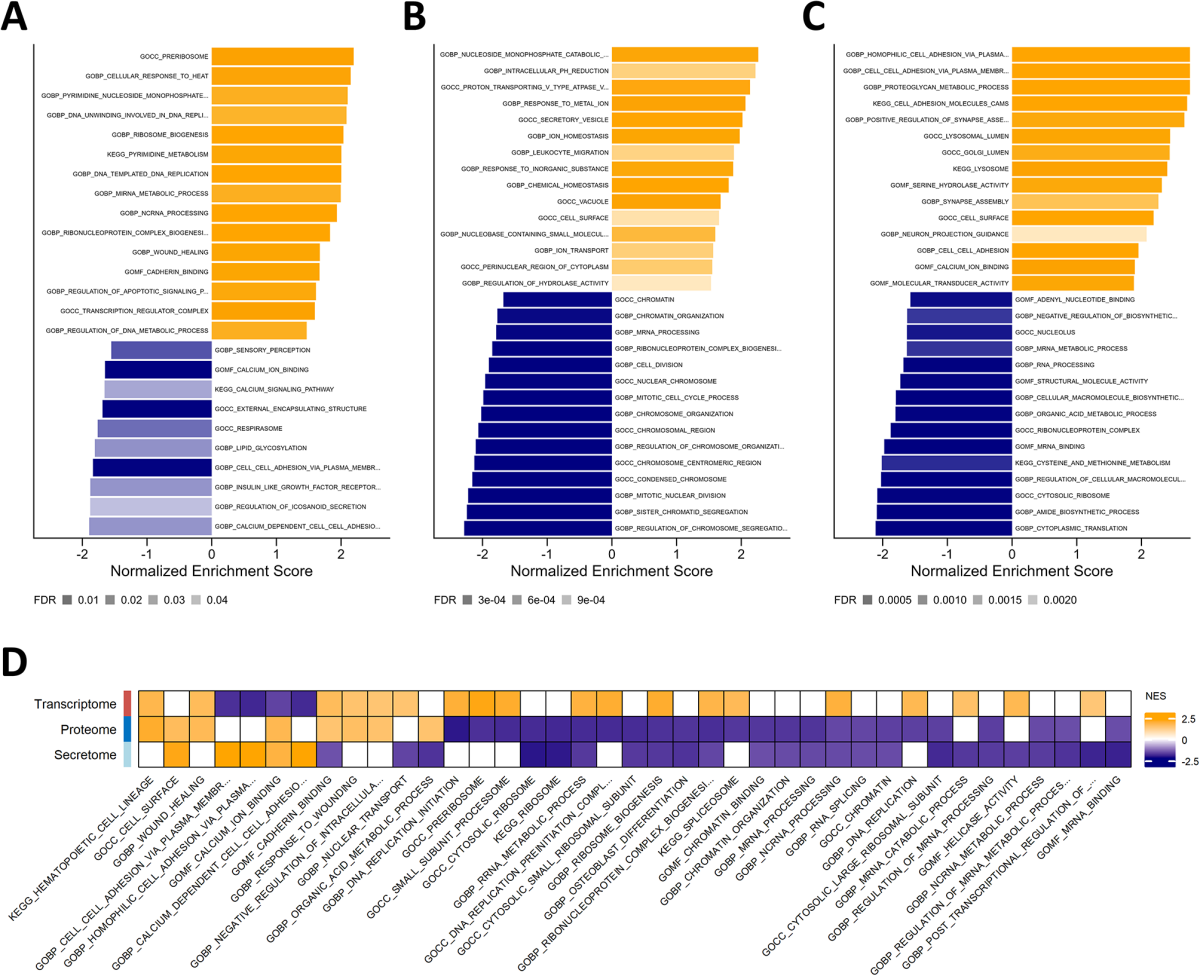
Gene set enrichment analysis of mRNAs and proteins with expression altered by *CDR2L* knockout. **A-C**, The top 30 (sorted by significance) up- and downregulated GO and KEGG gene sets in **(A)** transcriptome, **(B)** proteome, and **(C)** secretome of *CDR2L*-knockout cells. The gene sets were collapsed to reduce redundancy in the results. The horizontal axis represents the positive (orange) and negative (blue) normalized enrichment scores. The transparency of the bars reflects significance (at FDR < 0.05). **(D)** Heatmap of enriched pathways common to at least two datasets. The columns correspond to enriched GO and KEGG pathways, rows correspond to omic datasets. The colour and transparency of each cell represents the direction of the normalized enrichment scores (NES) and the significance, respectively

To better understand the biological functions of the genes with expression altered by knockout of *CDR2L*, we performed gene set enrichment analysis. There were both common pathways and pathways specifically enriched in the transcriptome, proteome, and secretome. At the transcript level, the most significantly upregulated pathways were related to ribosomes, DNA replication, and RNA processing (Fig. 2A and Table S8). The few downregulated pathways included cell adhesion and calcium binding (Fig. 2A and Table S8). In the proteome, downregulated pathways were mostly related to the cell cycle, RNA processing, and ribosome biogenesis, while upregulated pathways were enriched for nucleoside metabolism, immune responses, homeostasis, and regulation of actin filaments (Fig. 2B and Table S8). Downregulated pathways in the secretome were mainly related to ribosomes, RNA processing, and amide biosynthesis, and upregulated pathways were related to adhesion, proteoglycan metabolism, lysosomes, and synapse assembly (Fig. 2C and Table S8).

Interestingly, several of the pathways upregulated in the transcriptome were downregulated in the proteome and/or secretome (Fig. 2D): These pathways were related to RNA processing, DNA replication, and ribosome biogenesis. On the other hand, cell adhesion and calcium binding were downregulated in the transcriptome but upregulated in the secretome. Enriched pathways in the proteome and secretome showed the same direction of change.

### Ribosome biogenesis factors are dysregulated in the transcriptome and proteome of *CDR2L*-knockout cells, but in opposite directions

**Fig. 3:**
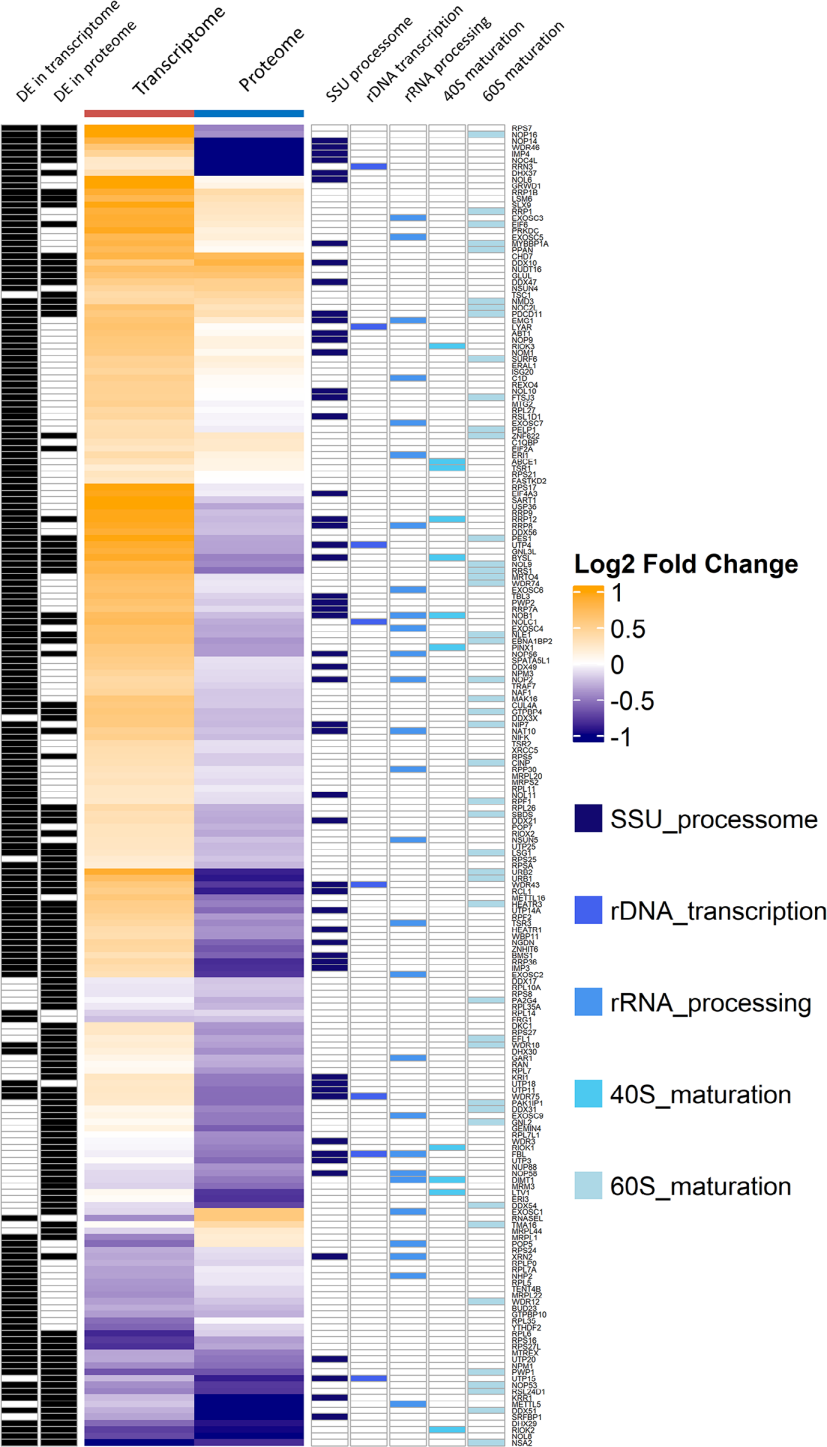
Ribosome biogenesis factors are affected differently in the transcriptome and proteome of *CDR2L*- knockout cells. Heatmap of differentially expressed genes from the GO term “ribosome biogenesis” detected in the transcriptome and proteome of *CDR2L*-knockout cells. The columns correspond to the datasets, and the rows correspond to individual genes. The colour of each cell represents the direction of the log2 fold change. The annotations on the left side of the heatmap describe whether the gene is significantly differently expressed, and the annotations on the right side indicate the step in the ribosome biogenesis pathway with which the gene is associated

CDR2L has previously been shown to localize to ribosomes and interact with ribosomal proteins [15]. As gene set analysis of *CDR2L*-knockout cells showed enrichment of ribosome biogenesis at the transcriptome and proteome level, we evaluated the expression of all genes with the GO term “ribosome biogenesis” significantly DE in at least one of the datasets (*n* = 206). In the transcriptome, the majority (80.4%) of DE mRNAs (*n* = 168) involved in ribosome biogenesis were upregulated, while in the proteome, the majority (84.2%) of DE proteins (*n* = 120) were downregulated. Eighty-one genes were DE in both transcriptome and proteome. Ribosome biogenesis is a complex process involving several highly orchestrated steps. To determine which steps were affected by the loss of CDR2L, we classified the genes according to a recent review of ribosome biogenesis factors [18]. In both the transcriptome and the proteome, most of the DE genes were associated with the small subunit processome, maturation of the 60S subunit, and ribosomal RNA (rRNA) processing (Fig. 3). That the same steps of ribosome biogenesis are affected in both transcriptome and proteome, albeit in opposite directions, strengthens the hypothesis that CDR2L is intimately involved in ribosomal function.

Dysregulated ribosome biogenesis interferes with the assembly of functional ribosomes and consequently inhibits protein translation. Therefore, we investigated the expression of individual ribosomal proteins and eukaryotic initiation factors (eIFs) that are essential for initiation of translation. In the transcriptome of *CDR2L*-knockout cells, genes encoding 39 ribosomal proteins were DE; of these 24 were downregulated. In the proteome and secretome, 37 and 53 ribosomal proteins were DE, respectively, of which all were downregulated. We also observed dysregulation of eIFs. A number were upregulated in the transcriptome, including EIF3B, EIF4A3, EIF5A, EIF5B, and EIF6, while EIF4G3, EIF4B, and EIF3K were downregulated. In the proteome, eIFs EIF1, EIF3L, EIF4B and EIF4G3 were downregulated. In the secretome, all DE eIFs were downregulated. These changes suggests that loss of CDR2L disrupts protein synthesis in the cells.

### Knockout of *CDR2L* causes downregulation of key regulators of the cell cycle

**Fig. 4:**
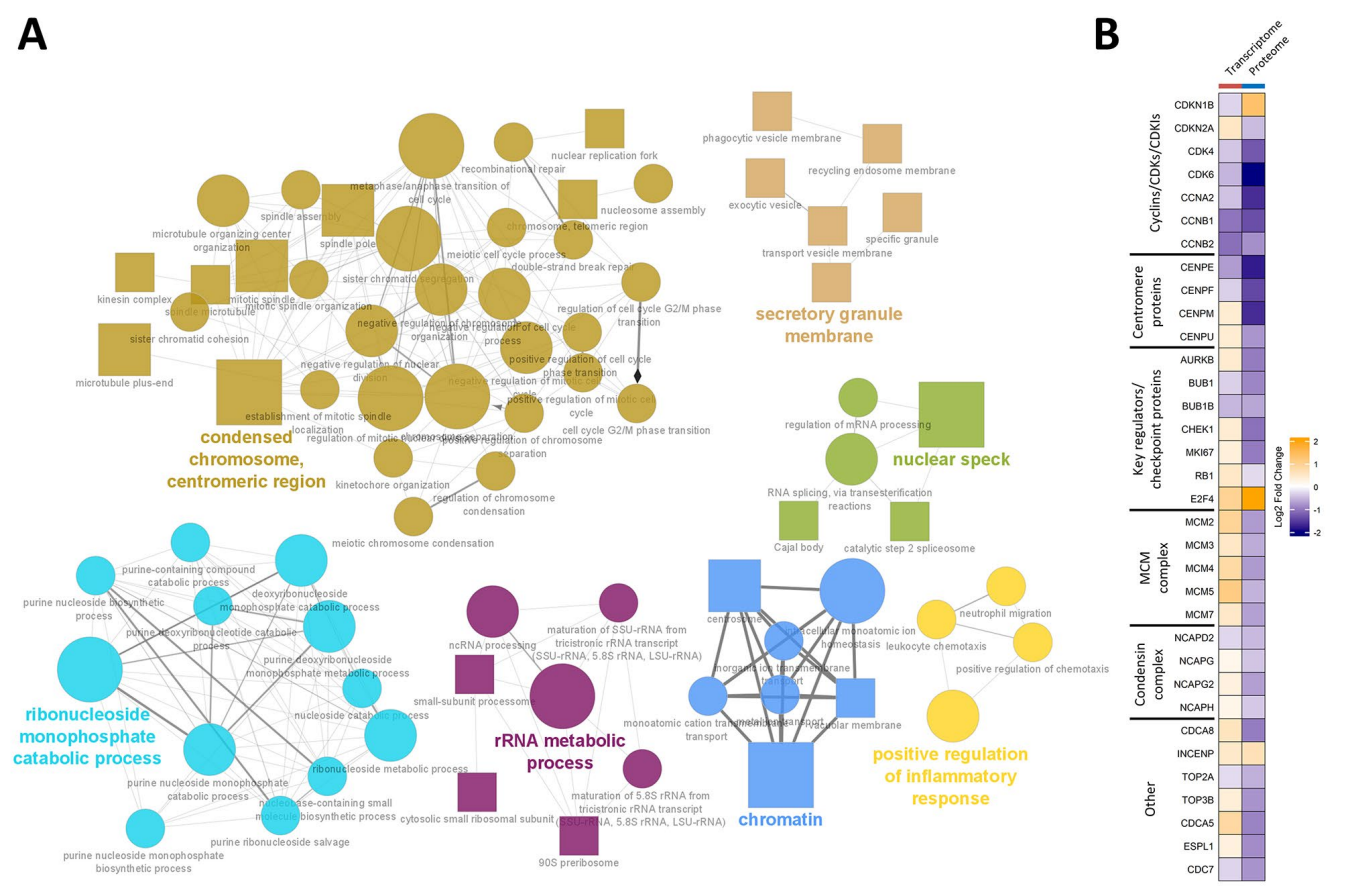
Knockout of *CDR2L* affects cell cycle-related pathways. **(A)** GO terms from the gene set enrichment analysis of the proteome of *CDR2L*-knockout cells visualized as a functionally organized network. Node colours represent grouped pathway terms. Group titles were chosen by selecting the most significant term in that group. Node shape represents the database source: circle, GO biological process; hexagon, GO molecular function; and square, GO cellular component. Significance of each pathway is reflected by the node size, with a larger shape representing a higher significance. Term-term interactions are shown as edges in the network where the thickness of the edge represents the extent of overlap. Only groups with more than two nodes are shown. **(B)** Heatmap of selected cell cycle-related genes significantly differentially expressed (FDR < 0.05) in both transcriptome and proteome of *CDR2L*-knockout cells. The columns correspond to the datasets, and the rows correspond to individual genes. The colour of each cell represents the direction of the log2 fold change. CDKs, cyclin-dependent kinases; CDKIs, cyclin-dependent kinase inhibitors; MCM, minichromosome maintenance

Gene set enrichment analysis of *CDR2L*-knockout cells resulted in more enriched pathways for the proteome (*n* = 325) than for the transcriptome (*n* = 61) or secretome (*n* = 139). The large number of pathways in the proteome rendered interpretation difficult. Therefore, we created a term network to investigate the functional relationships between these pathways. Most of the identified functional groups were related to the cell cycle, including cell cycle phase transition, mitotic spindle organization, chromatid segregation, and nuclear division (Fig. 4A). Other enriched pathways were related to rRNA processing and nucleoside metabolism – processes that are tightly connected to the cell cycle. Pathways related to the cell cycle and rRNA processing were all downregulated, whereas pathways related to nucleoside metabolism were upregulated in cells lacking CDR2L.

In relation to this finding, analysis of individual genes showed that knockout of *CDR2L* caused altered expression of multiple important regulators of the cell cycle in the proteome and/or transcriptome, including genes encoding Aurora kinases AURKA and AURKB, checkpoint regulators (BUB1, BUB1B, CHEK1, and RB1), cyclins (CCNA2, CCNB1, and CCNB2), cyclin-dependent kinases (CDK2, CDK4, and CDK6), cell division cycle proteins (CDC7, CDC20, CDC23, and CDC27), and cyclin-dependent kinase inhibitors (CDKN1A, CDKN1B, and CDKN2A). Other dysregulated proteins included components of the minichromosome maintenance protein complex, which is essential for DNA replication (MCM2, MCM3, MCM4, MCM5, and MCM7), subunits of the condensin complex, which plays a central role in chromosome condensation (NCAPD2, NCAPD3, NCAPG, NCAPG2, and NCAPH), centromere proteins, which are involved in chromosome segregation (CENPE, CENPF, CENPM, and CENPU), and several transcription factors (E2F, FOS, JUN, and MYC). Similar to ribosome biogenesis factors, several of the cell cycle-related genes were upregulated in the transcriptome but downregulated in the proteome (Fig. 4B). Proapoptotic factors and key mediators of apoptosis like BAD, BAX, BID, and PYCARD were upregulated in the *CDR2L*-knockout proteome. Taken together, these data suggest that loss of CDR2L suppresses cell cycle progression in ovarian cancer, which may trigger apoptosis.

Utilizing transcription factor enrichment analysis to identify the likely contribution of transcription factors to the changes in the transcriptome following loss of CDR2L, we identified several transcription factors which regulate the cell cycle and proliferation (Table S9). These included MYC, ERF, PA2G4, and subunits of the AP-1 transcription factor (FOSL1, FOSL2, and JUN) which regulate AURKB, BAX and several eIFs, among others, and members and interaction partners of the E2F family of transcription factors (E2F1, E2F4, E2F7, and TFDP1) which regulate CDK4 and CDK6. FOSL1 showed a particularly large upregulation in the transcriptome (log2 fold change = 4.4).

### CDR2L interacts with ribosome biogenesis factors and regulators of the cell cycle

In our earlier study, we used co-immunoprecipitation coupled with mass spectrometry to identify potential interaction partners of CDR2L in OVCAR-3 cells [15]. In light of our current findings, we re-examined our unpublished results and found that several of the dysregulated ribosome biogenesis factors were among the identified interaction partners of CDR2L including HEATR1, GTPBP4, PA2G4, PDCD11, SBDS, and UTP20, nucleolar proteins (NOP56 and NOP58), RNA helicases (DDX17, DDX21, DDX3X, and DHX29), and WD repeat-containing proteins (WDR18, WDR36, and WDR43). Several eIFs (e.g., EIF4G2, EIF2A, EIF3L, EIF6), cyclin-dependent kinases (CDK4 and CDK6), and components of the minichromosome maintenance protein complex (MCM2, MCM3, MCM4, MCM5, and MCM7) were also identified as potential interaction partners of CDR2L (Table S10). This strengthens the relationship between CDR2L and important regulators of ribosome biogenesis, protein synthesis and cell cycle progression.

### Ribosome-associated genes are key hubs in the network of genes differentially expressed in both transcriptome and proteome of *CDR2L*-knockout cells

**Fig. 5:**
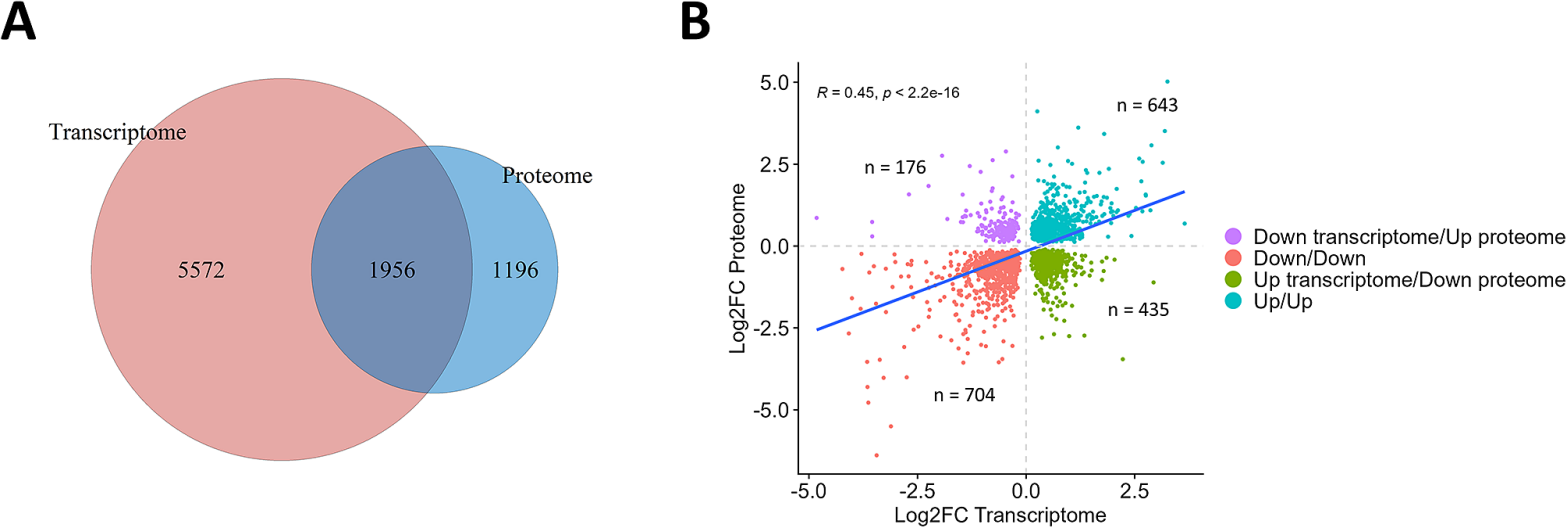
Comparison and correlational analysis between the transcriptome and proteome of *CDR2L*-knockout cells. **(A)** Venn diagram showing the number of shared and unique differentially expressed genes between the transcriptome and proteome of *CDR2L*-knockout cells. **(B)** Scatter plot showing log2 fold change of all genes significantly differentially expressed (FDR < 0.05) in both transcriptome (x-axis) and proteome (y-axis; *n* = 1958). The colour represents the four groups of possible combinations of direction of change. Correlation between log2 fold changes in transcriptome and proteome was assessed using Spearman’s rank correlation

We identified 1958 genes that were altered in both the transcriptome and the proteome of the *CDR2L*-knockout cells (Fig. 5A). Although most of these genes showed a consistent pattern of regulation, that is they were upregulated or downregulated in both datasets, 31.2% of the genes were oppositely altered in the transcriptome versus the proteome (Fig. 5B). This proportion was twice as high in the *CDR2L*-knockout cells as in the other *CDR*-knockout cells (Figure S2).

The genes with altered expression in both transcriptome and proteome of *CDR2L*-knockout cells were used to create a protein-protein interaction network using the STRING database. It consisted of a total 1958 nodes and 2745 edges. The genes in the network were enriched for many essential cellular processes including organelle organization, cell cycle and cell division, ribonucleoprotein complex biogenesis, gene expression, and intracellular transport (Table S11). By ranking the genes using the maximal clique centrality algorithm (see methods), we identified key hub genes with high connectivity within the network. The top 10 hub genes were all ribosome-associated genes including small ribosomal subunit proteins (RPS7 and RPS9), components of the small subunit processome (UTP4, UTP11, UTP20, HEATR1, WDR46, NOP56, and KRR1), and a nucleolar complex protein (NOC4L), again highlighting the effect of *CDR2L* knockout on ribosomal pathways and their interaction partners both at transcript and protein levels.

**Fig. 6:**
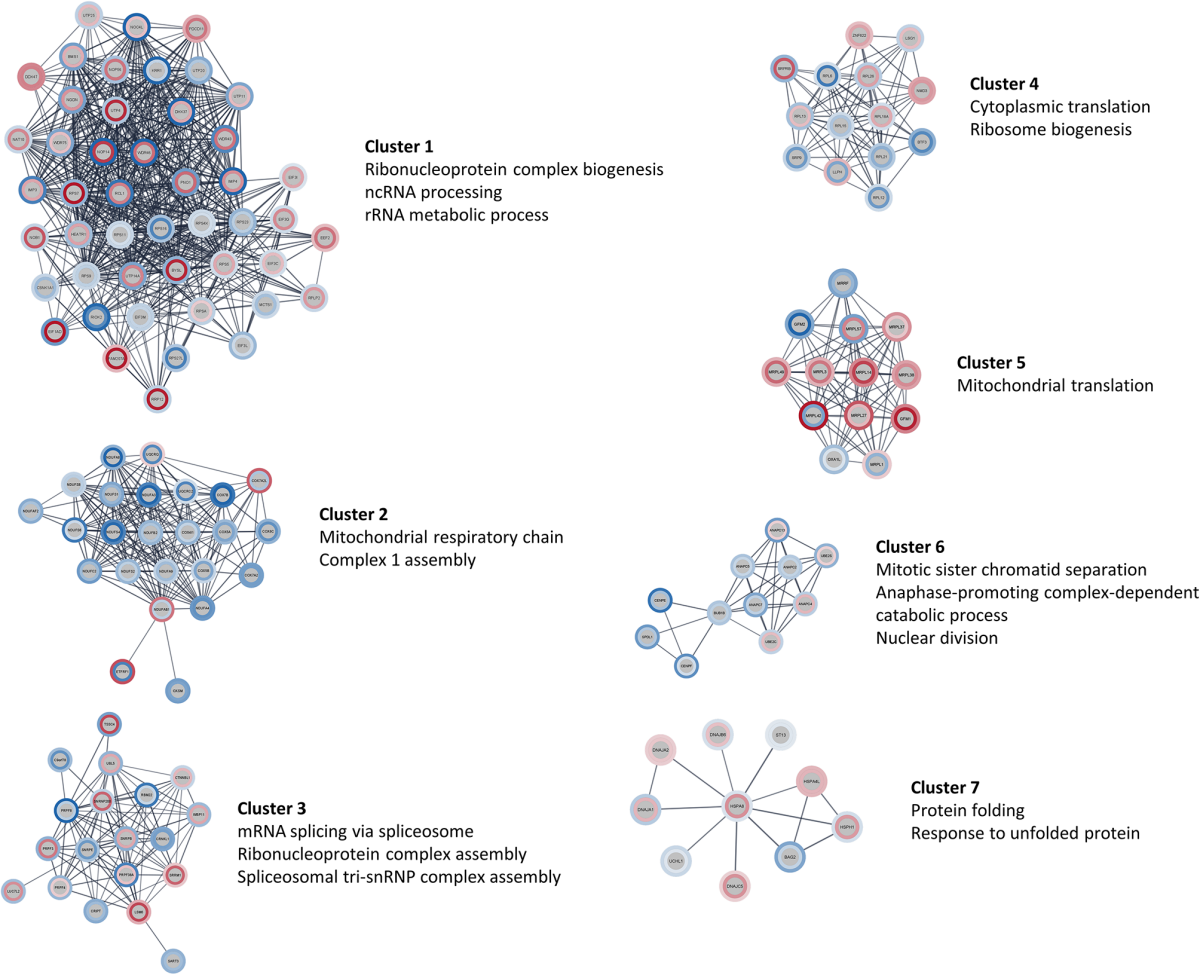
Protein-protein interaction networks of genes altered in both transcriptome and proteome of *CDR2L*-knockout cells. Genes significantly differentially expressed (FDR < 0.05) in both the transcriptome and proteome of *CDR2L*-knockout cells were used to create a protein-protein interaction network in STRING. The network was subsequently clustered using Markov clustering to identify clusters of tightly connected genes. Each node represents a gene, and the colour indicates down- (blue) or upregulation (red). Inner rings represent transcriptome data and outer rings represent proteome data. Clusters with > = 10 nodes are shown along with their most significant enriched GO biological process terms (FDR < 0.05)

Next, to identify clusters of tightly connected genes within the network, we performed Markov clustering analysis followed by enrichment analysis of the genes in the clusters. Three of the clusters showed enrichment for pathways related to ribosome biogenesis, RNA processing and translation: clusters 1, 3, and 4 (Fig. 6). The largest cluster (cluster 1) contained several components of the small subunit processome (UTP4, UTP11, UTP20, UTP25, NOC4L, NOP56, KRR1, and WDR46), RNA helicases (DDX47 and DHX37), translation initiation factor 3 subunits (EIF3I, EIF3G, EIF3C, and EIF3L), and small ribosomal subunit proteins (RPSA, RPS4X, RPS5, RPS9, RPS11, RPS16, and RPS23). Other clusters were distinctly enriched for mitochondrial pathways. Cluster 2 contained several subunits of complex 1 of the mitochondrial respiratory chain, which were downregulated in both transcriptome and proteome. Cluster 5 contained several mitochondrial ribosomal proteins, which were mostly upregulated in both datasets. Cluster 7 was enriched for protein folding. Taken together, these data indicate that loss of CDR2L results in dysregulation of protein synthesis and mitochondrial function.

### *CDR2L* knockout suppressed proliferation of ovarian cancer cells

**Fig. 7:**
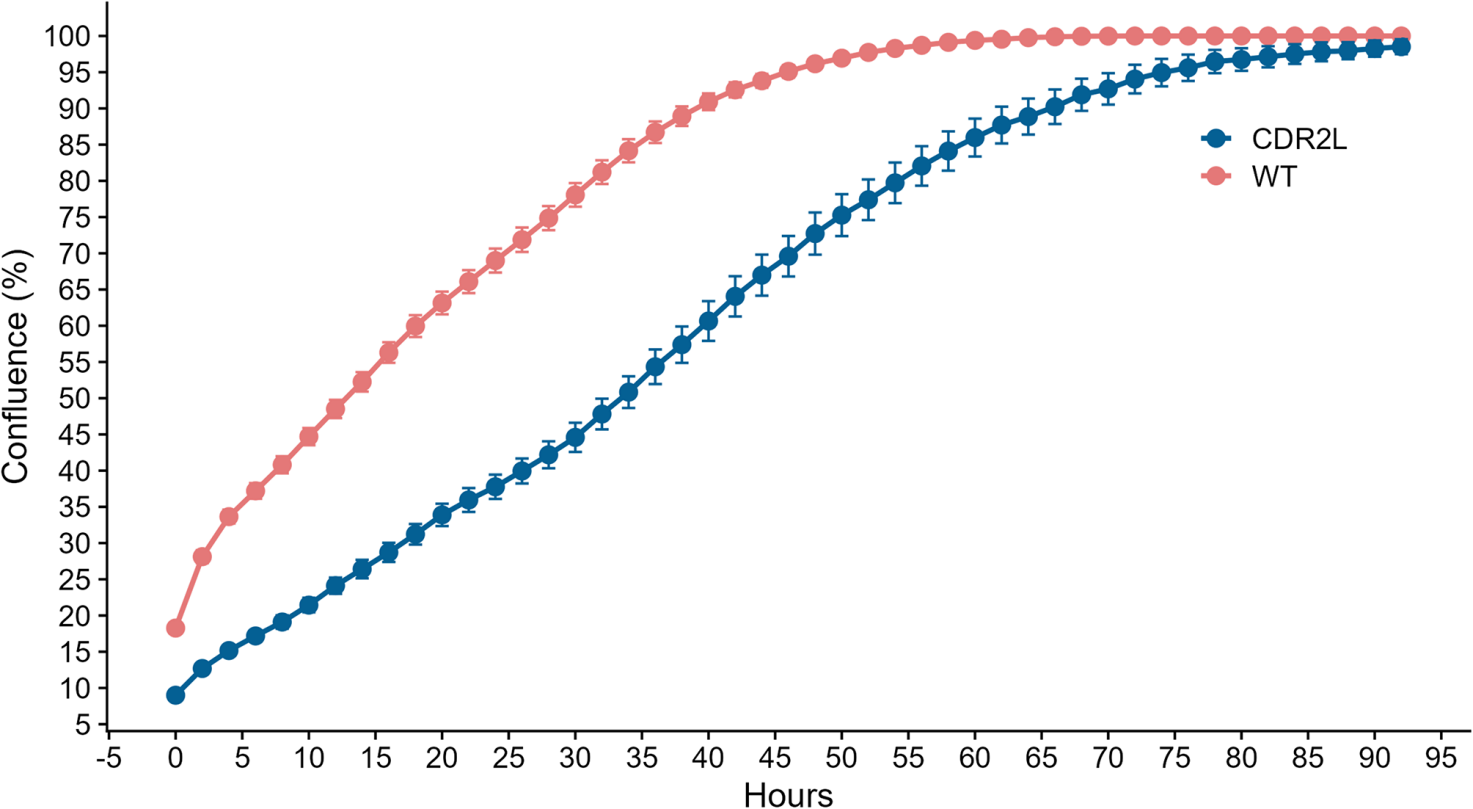
Analysis of WT and *CDR2L*-knockout ovarian cancer cell proliferation in vitro. The proliferation rate of wild-type (WT) OVCAR-3 cells and *CDR2L*-knockout cells was evaluated by live cell imaging. Plotted is the time in hours (x-axis) versus confluency calculated as a percentage of the surface of the imaged wells (y-axis). The data is presented as means with standard errors

To study the effect of *CDR2L* knockout on proliferation rates in vitro, we performed live cell imaging of WT cells and *CDR2L*-knockout cells. The WT cells reached 90% confluence after approximately 40 h, whereas *CDR2L*-knockout cells did not reach the same level of confluence until after approximately 65 h (Fig. 7). This demonstrates that *CDR2L* knockout markedly impaired cell proliferation in OVCAR-3 cells.

## Discussion

The pathological mechanisms underlying PCD are largely unknown. Tumour expression and genetic alterations of the CDR proteins, particularly CDR2L, is thought to trigger an immune response which targets both the cancer cells and the Purkinje neurons endogenously expressing the CDR proteins [19]. However, the mechanisms of immune activation and neural injury induced by these intracellular neural proteins remain unknown. This is in part due to our limited understanding of the biological properties of the CDR proteins. Here, we used transcriptomics and proteomics to evaluate the effects of CRISPR/Cas9-mediated knockout of CDR-encoding genes in ovarian cancer cells. As the CDR2L protein is likely to be the major target of the onconeural anti-Yo antibody in PCD [11], we performed an in-depth characterization of the *CDR2L*-knockout cells. Previously, we demonstrated that CDR2L colocalizes with ribosomes in ovarian cancer cells and Purkinje neurons [15]. Here, we show that loss of CDR2L causes dysregulation of ribosome biogenesis and reduces cell proliferation.

Changes in mRNA levels are generally assumed to be reflected by similar changes in the abundances of the corresponding proteins [20]. However, we found only a moderate correlation between protein abundances and mRNA levels, in line with previous studies [21, 22]. Therefore, integrating transcriptomic data with proteomic data provided us with a more comprehensive view of the cellular changes induced by knockout of *CDR2L*. Interestingly, we found that nearly one third of genes with expression altered at both transcriptomic and proteomic levels showed opposite trends of regulation, with the majority upregulated in the transcriptome and downregulated in the proteome. The proportion of DE genes with opposite regulation was twice as high in the *CDR2L*-knockout cells as in the other knockout cells, suggesting that CDR2L specifically affects the balance of mRNAs and proteins in the cell. Multiple processes contribute to this balance, including modulation of the translation rate by RNA-binding proteins, degradation of transcripts through binding of microRNAs, protein degradation through the ubiquitin-proteasome pathway or autophagy, temporal delay of protein synthesis, and availability of ribosomes in controlling the abundance of proteins [23]. Our work confirms that transcript abundance is not the only determinant of protein abundance, and highlights the importance of post-transcriptional, translational, and degradation regulation in controlling the abundance of proteins.

Early studies using serum-derived antibodies from PCD patients localized the Yo antigen to ribosomes and rough endoplasmic reticulum [13, 14], thereby providing the first indications that CDR2L, the major antigen of anti-Yo antibodies [11], was a regulator of protein synthesis. Using both Yo antibodies and commercial CDR2L antibodies, we confirmed the localization of CDR2L to ribosomes and identified the ribosomal protein RPS6 as a potential interaction partner of CDR2L [15]. In the present study, we found that knockout of CDR2L caused dysregulation of expression of multiple ribosome biogenesis factors, RNA processing factors, ribosomal proteins, and eukaryotic translation initiation factors. The majority of these were upregulated in the transcriptome and downregulated in the proteome and secretome. Downregulation of ribosome biogenesis factors, ribosomal proteins, and translation initiation factors should lead to reduced protein synthesis, with global effects favouring decreased protein abundances. In *CDR2L*-knockout cells, we identified similar numbers of increased and decreased mRNAs, but 1.5-fold more downregulated than upregulated proteins.

Upregulation of mRNAs involved in ribosome biogenesis, rRNA processing, and protein translation could be a consequence of attempts by cells to compensate for loss of ribosomal function and reduced protein synthesis. In support of this hypothesis, Myc, a proto-oncogenic transcription factor and an important regulator of ribosome biogenesis and cell proliferation [24], was upregulated in the transcriptome of *CDR2L*-knockout cells. Interestingly, previous studies have shown that CDR2 and c-Myc interact to synergistically regulate c-Myc-dependent transcription during passage through mitosis, and CDR2 was found to interact with and downregulate c-Myc function [25, 26]. As PCD sera blocked this interaction, it was suggested that increased c-Myc activity could contribute to the degeneration of Purkinje neurons seen in PCD. As these studies used a combination of antibodies from PCD patients and commercial anti-CDR2 antibodies, it is possible that some of these findings can be attributed to CDR2L. Further investigations into the CDR2L-Myc relationship are therefore warranted.

Several ribosome biogenesis factors, ribosomal proteins, and eukaryotic translation initiation factors were identified in the secretome of the OVCAR-3 cells, many of which were downregulated in the *CDR2L*-knockout cells. Although generally considered intracellular proteins, these can be found in extracellular vesicles (EVs) [27]. Ribosomal proteins are selectively incorporated into EVs and released from both normal and malignant cells [28]. The amounts and types of ribosomal proteins in EVs are altered under pathological conditions. For example, Dabbah et al. reported increased levels and repertoire of ribosomal proteins in EVs from mesenchymal stem cells in multiple myeloma compared with EVs from healthy controls [29]. These EVs were internalized by neighbouring cells and promoted their proliferation – a processes that was dependent on the ribosomal content of the EVs. The dysregulated ribosome biogenesis in *CDR2L*-knockout cells may therefore not only disrupt the activity of intracellular ribosomes but may also affect the levels and repertoire of ribosomal proteins released from cells in EVs, which in turn may affect the behaviour of neighbouring cells.

Ribosome biogenesis influences the cell cycle as it regulates cell size and growth. Disruption of ribosome biogenesis causes ribosomal stress, which results in p53-dependent cell cycle arrest and apoptosis [30]. In the present study, we showed that multiple pathways related to the cell cycle were downregulated in *CDR2L*-knockout cells. Important regulators of the cell cycle, such as cyclins (CCNA2, CCNB1, and CCNB2), cyclin-dependent kinases (CDK2, CDK4, and CDK6), and checkpoint regulators (BUB1, BUB1B, and CHEK1), were downregulated, whereas proapoptotic factors (BAD, BAX, and BID) were upregulated. CDK6, which together with CDK4 and cyclin D facilitate the progression of cells through the early G1 phase of the cell cycle [31], was highly downregulated (log2 fold change = -3.5). In line with downregulation of cell cycle proteins, knockout of *CDR2L* impaired cell proliferation in vitro. Similarly, reduction of levels of the ribosomal protein RPS6 in ovarian cancer cells caused downregulation of CDK2, CDK4, CDK6, cyclin E, and cyclin D1, thereby blocking the transition from G0/G1 to S phase and suppressing cell proliferation [32]. Previously, we showed that CDR2L interacts with RPS6 [15]. The observation that loss of either CDR2L or RPS6 causes similar effects on the cell cycle in ovarian cancer cells further supports an association between the two proteins. However, the effect of knockout of *CDR2L* on the cell cycle seems to be independent of changes in RPS6 expression as RPS6 was not differentially expressed following the knockout. Rather, knockout of *CDR2L* may alter the function of RPS6 or disrupt common downstream signalling events.

The current study provides an extensive bioinformatic analysis of the effect of *CDR*-knockout which can serve as a valuable resource for designing future experiments to determine the biological properties of the CDR proteins. For example, the effect on the cell cycle should be further explored using flow cytometry which would provide insight into which phases of the cell cycle is affected by the absence of the CDR2L protein. Rescue experiments in which CDR2L is re-expressed would provide further validation of the observed knockout phenotype. Comparative studies with all *CDR*-knockout cells would determine whether the effect on the cell cycle is unique to CDR2L, or whether it is a shared effect among the CDR proteins. Further, an orthotopic xenograft model could be used to explore the effects of *CDR*-knockout on the tumorigenic potential of ovarian cancer cells, including assessment of tumour growth rate and metastasis.

## Conclusions

In conclusion, we found that knockout of *CDR2L* in an ovarian cancer line dysregulates genes involved in ribosome biogenesis, protein synthesis and cell cycle-related processes, ultimately impairing cell proliferation. Yo antibodies from PCD patients are internalized by Purkinje neurons where they bind to CDR2L [9, 11]. Whether binding of anti-Yo to CDR2L interferes with CDR2L and thereby disrupts ribosome homeostasis or protein synthesis in Purkinje neurons remains to be explored. Deficits in ribosome biogenesis in neurons have been shown to cause dendritic degeneration and loss of synaptic plasticity [33]. The data provided by this study serves as a valuable resource for exploring the biological properties of the CDR proteins.

## Materials and methods

### Generation of knockout OVCAR-3 cells

*CDR1*-knockout, *CDR2*-knockout, and *CDR2L*-knockout OVCAR-3 cell lines and WT (#YC-D019) cells were provided by Ubigene Biosciences Co., Ltd. The single guide RNAs (sgRNAs) were designed using the online CRISPR design tool (https://en.rc-crispr.com/). The pair of oligonucleotides corresponding to each sgRNA were annealed and ligated into the YKO-RP003 vector (Ubigene Biosciences Co., Ltd.). The YKO-RP003-[sgRNA] plasmids were transfected into cells with Lipofectamine 3000 (Thermo Fisher Scientific). At 24–48 h after the transfection, puromycin was added. After antibiotic selection, cells were diluted by limited dilution methods and inoculated into 96-well plate. Selection of single clones was performed after 2–4 weeks, and knockout was validated by polymerase chain reaction and Sanger sequencing.

### Cell culture

OVCAR-3 cells were cultured in RPMI 1640 medium supplemented with 20% fetal bovine serum (Thermo Fisher Scientific) and 1% penicillin/streptomycin (Thermo Fisher Scientific) in a humidified atmosphere with 5% CO2 at 37 °C. Cells were used for experiments within 10 passages of thawing. Cells were routinely tested for mycoplasma (MycoAlert PLUS Mycoplasma Detection Kit).

### Cell proliferation assay

WT and *CDR2L*-knockout OVCAR-3 cells were seeded at 10,000 cells/well in Incucyte Imagelock 96-well plates (Sartorius). Following cell seeding, plates were placed in an Incucyte live-cell analysis system (Sartorius) and imaged every 2 h for 96 h. The rate of cell proliferation was determined based on changes in confluency of each imaged well. Analysis was performed using the Incucyte S3 software (Sartorius). The experiment was repeated three times with similar results.

### RNA sequencing

Total RNA was extracted from all four cell lines in biological triplicates using miRNeasy Tissue/Cells Advanced (Qiagen) according to the manufacturer’s protocol. Library preparation and sequencing was performed by BGI Genomics (https://www.bgi.com/global/). Briefly, RNA quality and concentration was assessed using the Agilent 2100 Bioanalyzer. mRNA was enriched using oligo (dT)-functionalized magnetic beads, fragmented, converted to double-stranded cDNA, and amplified using PCR. Samples were sequenced through combinatorial probe-anchor synthesis on the DNBSEQ sequencing system using paired-end 100-bp sequencing. Raw reads were filtered using SOAPnuke version 2.1.3. Reads containing the adaptor, reads with N content greater than 5%, and low-quality reads were removed.

### RNA expression quantification

Salmon version 1.10.2 [34] was used to quantify the abundance at the transcript level against the Ensembl release 75 transcriptome. Transcript-level quantification was collapsed onto gene-level quantification using the tximport R package version 1.26.1 [35]. Genes with zero counts and zero variance, genes without gene symbol, and mitochondria-encoded genes were excluded. Lowly expressed genes were excluded by only retaining genes where the counts across samples in the 80th percentile were greater than 0.1 counts per million. Differential gene expression analysis was performed using the DESeq2 R package version 1.32.2 [36] with default parameters. Multiple hypothesis testing was performed with the default automatic filtering of DESeq2 followed by FDR calculation by the Benjamini-Hochberg procedure.

### Liquid chromatography and mass spectrometry analysis

Whole cell lysate and conditioned cell media (CCM) was prepared from all four cell lines in biological triplicates and analyzed by mass spectrometry-based proteomics. Cells were lysed with RIPA lysis buffer (G-Biosciences) supplemented with protease inhibitors (Thermo Fisher Scientific) on ice for 15 min. Protein lysates were collected by centrifugation at 14,000 x g for 15 min at 4 °C. Protein concentration was measured with the BCA assay (Thermo Fisher Scientific). For CCM, cells cultured in T175 flasks (Corning) were incubated with media without any additives for 24 h. The CCM was collected, centrifuged at 3000 x g for 15 min and filtered through a 0.2-µm syringe filter (Thermo Fisher Scientific). The CCM was concentrated using Ultra-15 3-kD cut-off centrifugal filters (Millipore) and centrifugation at 3000 x g for 1 h.

The samples were mixed with paramagnetic beads (Sera-Mag Speed beads, GE Healthcare) in a 1:10 ratio. A 100% ethanol to 70% ethanol solution was added to the peptide-bead mixture, followed by agitation at 1000 rpm for 7 min at room temperature. The beads were washed twice in 80% ethanol to remove the lysis buffer, and 50 µl digestion buffer containing 100 mM AmBic, 1 mM CaCl_2_, and 0.2 µg/µl trypsin was added to each sample, followed by sonication for 30 s. Samples were incubated for 16 h at 37 °C in a thermomixer at 1,000 rpm. After digestion, the samples were centrifuged at 13,000 rpm at 24 °C for 3 min. Following magnetic separation, the supernatants were transferred to a fresh tube. NaCl (0.5 M) was added to the magnetic beads, the sample was sonicated for 30 s and centrifuged a second time at 13,000 rpm at 24 °C for 3 min. The supernatants of these tubes were added to the tubes with the previous supernatant. Trifluoroacetic acid (TFA, 0.1%, 200 µl) was added to each sample.

Oasis 96-well cartridges (Waters) were used for desalting. Cartridges were activated by adding 500 µl of 80% acetonitrile (ACN), 0.1% formic acid (FA) and centrifuged at 200 x g for 1 min. The cartridges were then washed three times with 0.1% TFA. After adding the samples, the cartridges were centrifuged at 100 x g for 3 min. The flow through was discarded, and cartridges were washed twice with 0.1% TFA followed by a short centrifugation. Samples were eluted with 100 µl of 80% ACN, 0.1% FA. The samples were freeze dried prior to tandem mass tag labelling.

Approximately 0.5 µg protein as tryptic peptides dissolved in 2% ACN and 0.5% FA, were injected into an Ultimate 3000 RSLC system (Thermo Fisher Scientific) connected online to Orbitrap Eclipse mass spectrometer (Thermo Fisher Scientific) equipped with EASY-spray nano-electrospray ion source (Thermo Fisher Scientific). For trapping and desalting process, the samples were loaded and desalted on a pre-column (Acclaim PepMap 100, 2 cm × 75 μm ID nanoViper column, packed with 3 μm C18 beads) at a flow rate of 5 µl/min for 5 min with 0.1% TFA. Peptides were separated during a biphasic ACN gradient from two nanoflow UPLC pumps (flow rate of 250 nl/min) on a 25-cm analytical column (PepMap RSLC, 50 cm × 75 μm ID EASY-Spray column, packed with 2-µm C18 beads). Solvent A and B were 0.1% FA (vol/vol) in distilled H_2_O and 100% ACN, respectively. The gradient composition was 5% B during trapping (5 min) followed by 5–7% B over 30 s, 8–22% B for the next 145 min, 22–28% B over 16 min, and 35–80% B over 15 min. Elution of very hydrophobic peptides and conditioning of the column were performed during a 15 min isocratic elution with 90% B and a 20 min isocratic elution with 5% B, respectively. The peptides eluted from the LC-column were ionized in the electrospray and analyzed by the Orbitrap Eclipse. The mass spectrometer was operated in the data-dependent-acquisition mode to automatically switch between full scan MS and MS/MS acquisition. Instrument control was through Tune 2.7.0 and Xcalibur 4.4.16.14.

Survey full-scan MS spectra (from m/z 375 to 1500) were acquired in the Orbitrap with resolution *R* = 120,000 at m/z 200 after accumulation to a target value of 4e^5^ in the C-trap, ion accumulation time was set as auto. FAIMS was enabled using two compensation voltages (CVs), -45 V and − 65 V respectively. During each CV, the mass spectrometer was operated in data-dependent-acquisition mode to automatically switch between full scan MS and MS/MS acquisition. The cycle time was maintained at 0.9 s/CV. The most intense peptides with charge states 2 to 6 were sequentially isolated to a target value (AGC) of 2e^5^ and maximum IT of 120 ms in the C-trap, and isolation width maintained at 0.7 m/z. Fragmentation was performed with a normalized collision energy of 30%, and fragments were detected in the Orbitrap at a resolution of 30,000 at m/z 200, with first mass fixed at m/z 110. The spray and ion-source parameters were as follows: ion spray voltage = 1900 V, no sheath and auxiliary gas flow, and capillary temperature of 275 °C. The raw files were analyzed using Proteome Discoverer 2.4 (Thermo Fisher Scientific). Spectra were matched against the *Homo sapiens* database obtained from UniProt. The final protein intensity files were exported as txt files and used for differential expression analysis.

### Protein expression quantification

Protein intensity txt files were analyzed for differentially expressed proteins using R version 4.2.2. First, proteins labelled as “Contaminant” by Proteome Discoverer were removed from the analysis. Second, to be included for downstream analysis, proteins were required to have non-zero intensity in all replicates in at least one experimental group. The filtered proteomic dataset was composed of 7447 proteins. Missing values (*n* = 1830) were imputed using the missForest function from the missForest R package version 1.5 [37], which applies a random forest imputation algorithm shown to perform well with proteomics data [38]. Additionally, we removed proteins with a median abundance value less than the 5th percentile. To test for differential expression we used the lmFit and eBayes functions from the limma R package version 3.54.1 [39].

### Pathway enrichment analysis

Gene set enrichment analysis was performed using the fgsea R package version 1.24.0 [40]. The specific parameters used are documented in the code for the analysis. Gene sets derived from the GO [41] and the KEGG [42] databases were downloaded from the Molecular Signatures Database [43]. Gene sets with an FDR adjusted *p*-value < 0.05 were considered significant. Over-representation analysis of differentially expressed genes and proteins was performed using the WebGestaltR R package version 0.4.4 [44], with GO and KEGG gene sets. Gene sets with an FDR adjusted *p*-value < 0.05 were considered significant.

### Protein-protein interaction network

The protein-protein interaction network was generated based on the interactions derived from the STRING database using the Cytoscape STRING app version 2.0.1 [45] with a strict confidence score cutoff of 0.8 and network type set to “physical subnetwork”. The network was visualized using Cytoscape version 3.9.1 [46], and nodes in the network were coloured according to their log2 fold change using the Omics Visualizer app version 1.3.0 [47]. Key hub genes were identified using the maximal clique centrality algorithm in the CytoHubba app version 0.1 [48]. To cluster the network based on their interactions from STRING, we used the clusterMaker2 version 2.3.4 [49] and performed Markov clustering with default parameters. Enrichment analysis of the genes in the clusters was performed using the Cytoscape STRING app. Gene sets with an FDR adjusted *p*-value < 0.05 were considered significant.

### Pathway term network

Enriched pathways were grouped into functionally organized networks using ClueGO version 2.5.10 [50] and Cluepedia version 1.5.10 [51]. The networks were visualized using Cytoscape.

### Transcription factor enrichment analysis

Transcription factor enrichment analysis was performed by analyzing the differentially expressed mRNAs (FDR < 0.05) using the ChIP-X Enrichment Analysis version 3 (ChEA3) [52] with default parameters.

### Electronic supplementary material

Below is the link to the electronic supplementary material.

Supplementary Material 1. supplementary_table_S1.xlsx. Transcriptome dataset. Expression data provided as counts per million. Genes filtered for mitochondria-encoded genes and lowly expressed genes.

Supplementary Material 2. supplementary_table_S2.xlsx. Proteome dataset. Expression data provided as normalized abundance values. Proteins were filtered for contaminants and lowly expressed proteins.

Supplementary Material 3. supplementary_table_S3.xlsx. Secretome dataset. Expression data provided as normalized abundance values. Proteins were filtered for contaminants and lowly expressed proteins.

Supplementary Material 4. supplementary_table_S4.xlsx. Results of differential expression analysis of knockout CDR2L cells vs wildtype cells.

Supplementary Material 5. supplementary_table_S5.xlsx. Results of differential expression analysis of knockout CDR2 cells vs wildtype cells.

Supplementary Material 6. supplementary_table_S6.xlsx. Results of differential expression analysis of knockout CDR1 cells vs wildtype cells.

Supplementary Material 7. supplementary_table_S7.xlsx. Results of over-representation analysis of differentially expressed genes common across all knockout cell lines.

Supplementary Material 8. supplementary_table_S8.xlsx. Results of gene set enrichment analysis of knockout CDR2L cells vs wildtype cells.

Supplementary Material 9. supplementary_table_S9.xlsx. Results of transcription factor enrichment analysis of knockout CDR2L cells. Analysis was performed using ChEA3 and results are given as mean rank.

Supplementary Material 10. supplementary_table_S10.xlsx. Proteins identified as potential interaction partners of CDR2L using co-immunoprecipitation and mass spectrometry. Listed are the proteins along with the antibodies used for the co-immunoprecipitation.

Supplementary Material 11. supplementary_table_S11.xlsx. Results of enrichment analysis of co-differentially expressed genes from the STRING network.

Supplementary Material 12. supplementary_figure_S1.png. Scatterplots with associated Spearman’s rank correlation of gene expression between datasets. (A) RNA expression in counts per million (CPM; x-axis) and proteome protein expression as log2 transformed abundance values (y-axis). (B) RNA expression in CPM (x-axis) and secretome protein expression as log2 transformed abundance values (y-axis). (C) Proteome protein expression as log2 transformed abundance values (x-axis) and secretome protein expression as log2 transformed abundance values (y-axis).

Supplementary Material 13. supplementary_figure_S2.png. A-B, Scatter plots showing log2 fold change of all genes significantly differentially expressed (FDR < 0.05) in both transcriptome (x-axis) and proteome (y-axis) in knockout CDR1 cells (A) and knockout CDR2 cells (B). The colour represents the four groups of possible combinations of direction of change. Correlation between log2 fold changes in transcriptome and proteome was assessed using Spearman’s rank correlation.

## Data Availability

All data generated or analysed during this study are included in this published article and its supplementary files. The source code and all necessary files to perform the analyses are available on GitHub under the GPL public license v3.0: https://github.com/ETSunhome.
