# Supplementary figures and images for: Multi-omics profiling reveals dysregulated ribosome biogenesis and impaired cell proliferation following knockout of CDR2L
Source: BMC Cancer. 2024 May 27;24:645. doi: 10.1186/s12885-024-12399-z (PMC11129367; doi:10.1186/s12885-024-12399-z)

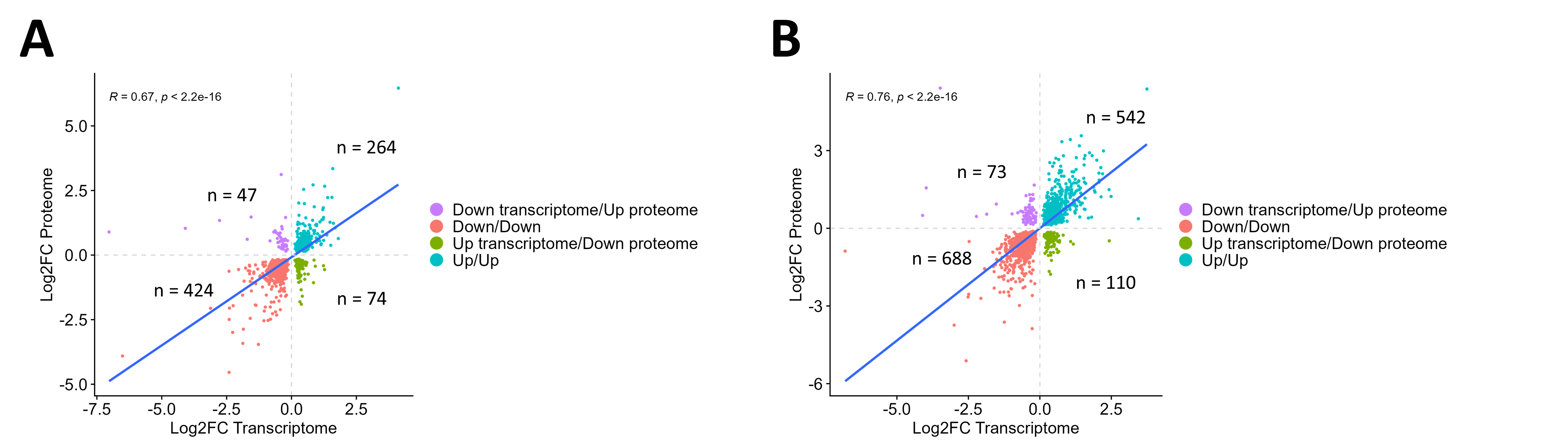

Supplement: Supplementary file 12 — Supplementary Material 12. supplementary_figure_S1.png. Scatterplots with associated Spearman’s rank correlation of gene expression between datasets. (A) RNA expression in counts per million (CPM; x-axis) and proteome protein expression as log2 transformed abundance values (y-axis). (B) RNA expression in CPM (x-axis) and secretome protein expression as log2 transformed abundance values (y-axis). (C) Proteome protein expression as log2 transformed abundance values (x-axis) and secretome protein expression as log2 transformed abundance values (y-axis). [file 12885_2024_12399_MOESM12_ESM.png]

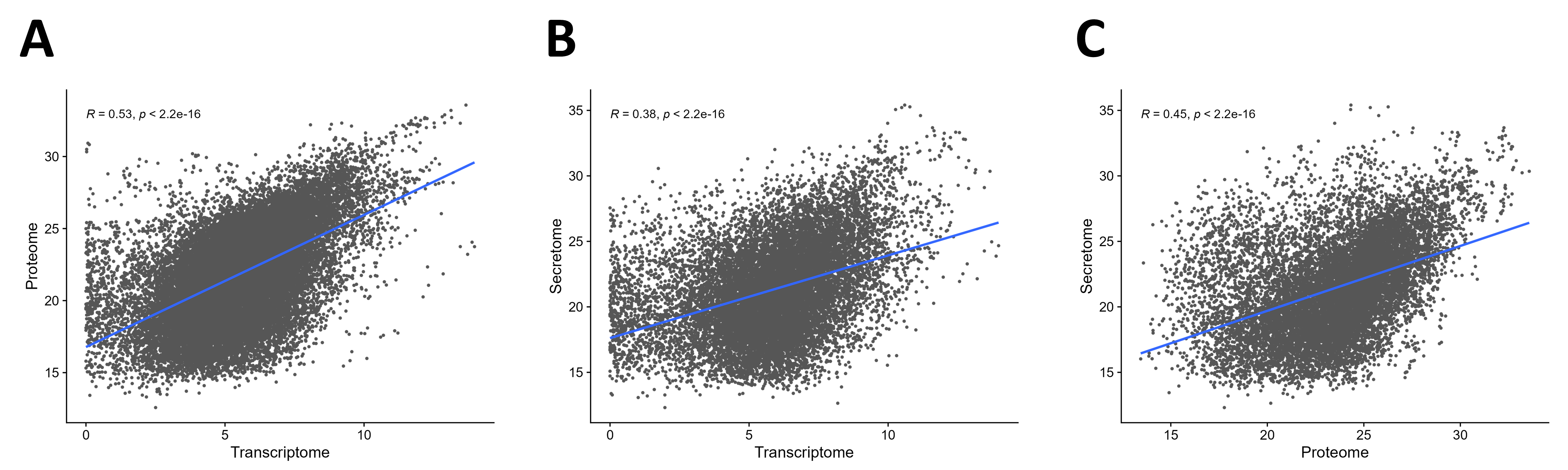

Supplement: Supplementary file 13 — Supplementary Material 13. supplementary_figure_S2.png. A-B, Scatter plots showing log2 fold change of all genes significantly differentially expressed (FDR < 0.05) in both transcriptome (x-axis) and proteome (y-axis) in knockout CDR1 cells (A) and knockout CDR2 cells (B). The colour represents the four groups of possible combinations of direction of change. Correlation between log2 fold changes in transcriptome and proteome was assessed using Spearman’s rank correlation. [file 12885_2024_12399_MOESM13_ESM.png]
